# Gender differences in cancer-related distress in Japan: a retrospective observation study

**DOI:** 10.1186/s13030-016-0062-8

**Published:** 2016-04-12

**Authors:** Atsuko Koyama, Hiromichi Matsuoka, Yoichi Ohtake, Chihiro Makimura, Kiyohiro Sakai, Ryo Sakamoto, Masahiko Murata

**Affiliations:** Department of Psychosomatic Medicine, Kinki University, Faculty of Medicine, 377-2, Ohno-higashi, Osakasayama City, Osaka 589-8511 Japan

**Keywords:** Cancer, Distress, Total pain, Gender differences, Gender-based medicine, Psycho-oncology

## Abstract

**Background:**

Cancer care is currently the most important medical issue in Japan. Total pain of cancer patients consists of a combination of four factors: physical, psychological, social distress, and spiritual pain. Previous studies showed female cancer patients ask for more psychological support and seem to suffer different types of distress compared with male patients, for example, appearance-related symptoms. However, other factors of cancer distress related to gender have not been defined comprehensively. The aim of this study is to clarify the gender differences in cancer distress types in order to elucidate the measures that should be taken in Japan to improve the quality of whole cancer care based on gender-based medicine.

**Methods:**

The data of new patients who had visited the psycho-oncology outpatient service of Kinki University Hospital during the period of May 2013 to October 2015 were collected. Demographic factors and all assessed items were extracted from the patients’ medical charts retrospectively. Based on an inquiry of cancer patients in 2010, each item representing the four factors of “total pain” of cancer patients was chosen, i.e., physical distress (pain, changes in appearance), psychological distress (anxiety, depression), social distress (family problems, job-related problems), and spiritual pain; together with sexuality issues, and answers were analyzed. Hospital Anxiety Depression Scale (HADS) was used for the assessment of psychological distress. Chi-square test and Fisher’s exact test were performed for gender differences in the cancer distress types. Pearson’s analysis and multiple logistic regression analysis were performed for the association of gender with each item.

**Results:**

The data of 101 cancer patients were analyzed and there were more female patients than male patients (female: male ratio = 71:30). Female cancer patients were more likely to suffer from psycho-social issues such as changes in appearance, family problems and sexuality issues than male patients, and male patients were more likely to have spiritual pain.

**Conclusions:**

There were gender differences in the distress types of cancer patients. In order to improve the quality of whole cancer care, more intensive intervention by medical professionals and social support is needed from the viewpoint of gender-based medicine and psycho-oncology.

## Background

For the last few decades, the prevalence of cancer has been increasing in Japan. Cancer has been the leading cause of death among Japanese people since 1981 and approximately one third of Japan’s population will die of cancer [1]. Thus, cancer care is currently the most important medical issue in Japan. In order to improve the quality of life of both cancer patients and their families, the Japanese government developed a plan for cancer care that has been used since 2004. The plan is revised every 5 years. The Cancer Control Act [2] was enforced in 2007 and states the importance of psychosocial care, as well as physical care for cancer patients. The complicated distress of cancer patients is called “total pain”, which was advocated by Cicely Saunders who created St. Christopher’s Hospice in England [3]. Total pain of cancer patients consists of a combination of four factors: physical, psychological, social distress, and spiritual pain.

Previous studies showed that more than 30 % of cancer patients have a need for psychosocial support [4–7] and that female patients requested more assistance [8]. There were role and gender differences regarding psychological distress and quality of life when dealing with cancer. Not only female cancer patients, but also female partners of male cancer patients, had psychological distress and a low quality of life [9, 10]. In addition, female cancer patients seem to suffer different types of distress compared with male patients. For example, women suffered from more appearance-related symptoms and distress resulting from chemotherapy than men [11]. However, other factors of cancer distress related to gender have not been defined comprehensively.

The aim of this study is to clarify the differences in distress types between female and male cancer patients in order to elucidate the measures that should be taken in Japan to improve the quality of whole cancer care from the viewpoint of gender-based medicine and psycho-oncology.

## Methods

### Patients

The department of psychosomatic medicine, Kinki University Faculty of Medicine operates a specific outpatient service for cancer patients. The data of new patients who had visited the specific outpatient service for psycho-oncology during the period of April 2013 to October 2015 were collected. All patients were at least 16 years old.

### Design and settings

All patients filled out a medical questionnaire and a semi-conducted interview was performed asking for more details based on the questionnaire during their first visit to our department, with the answers recorded in their medical charts.

**Fig. 1:**
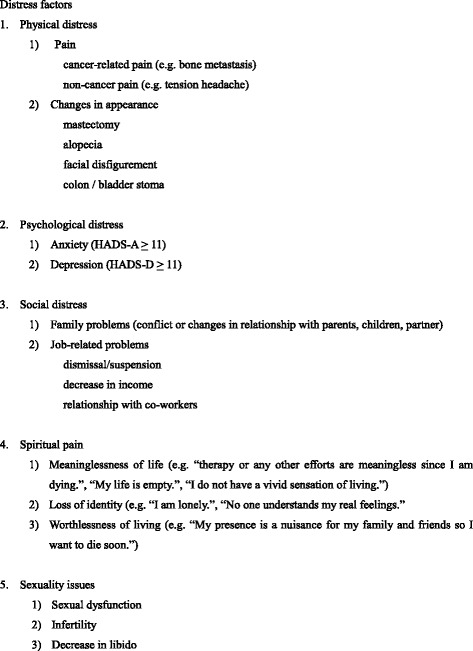
The criteria of items of cancer distress factors. Based on the inquiry of cancer patients in 2010 [12], each item representing the four factors of “total pain” of cancer patients, i.e., physical distress (pain, changes in appearance), psychological distress (anxiety, depression), social distress (family problems, job-related problems), and spiritual pain; along with sexuality issues, were chosen to be used in this study. All items were assessed using a binary scale: ‘yes’ or ‘no’. The criteria for designating an item as ‘yes’ is indicated in Fig. 1

All the items assessed were extracted from the patients’ medical charts retrospectively. Demographic factors such as age, gender, cancer site, period of time after first diagnosis or recurrence, and profiles of therapies before the assessment (operation, chemotherapy, radiation, hormonal therapy) were extracted from the medical charts. Based on the inquiry of cancer patients in 2010 [12], each item representing the four factors of “total pain” of cancer patients was chosen: Physical distress (pain, changes in appearance), psychological distress (anxiety, depression), social distress (family problems, job-related problems), and spiritual pain; together with sexuality issues and answers were analyzed. All items were assessed with a binary scale: ‘yes’ or ‘no’. In order to minimize an assessment bias among researchers, the criteria for designating an item as ‘yes’ is indicated in Fig. 1. The items of “pain” included both cancer and non-cancer pain, such as headache and abdominal pain, and were assessed using ‘yes’ or ‘no’. Examples of questions are: “Do you have cancer-related pain such as bone metastasis or non-cancer related pain such as tension headache? Which best describes your condition on the Numeric Rating Scale (NRS) (0–10) ?” The patients with some pain, except for those who chose 0 in NRS, were judged as ‘yes’. The items of “changes in appearance” included mastectomy, alopetia due to chemotherapy, facial disfigurement of the head and neck, and colon or bladder stoma. The items of “anxiety and depression” were assessed by the Hospital Anxiety Depression Scale (HADS) [13], which contains 14 questions and 4 degrees (0, 1, 2, 3) in each answer. All of the patients completed this psychological test for the assessment of psychological distress during their first visit to our department. In HADS, a cut-off score of 11 was adopted in this study to determine that patients were considered to be in an anxiety and/or depressive state respectively. The items of “family problems” included conflicts and/or changes in relationship with the patients’ parents, children, or partner. Examples of questions were: “Do you have any problems related to your family? With whom? What is your family member’s reaction?” Responses such as “I cannot take care of my parents anymore.”, “I am concerned about my children’s future.”, or “My partner is avoiding conversations about cancer and/or sex with me.” in the medical charts were counted as family problems. The items of “job-related problems” contained dismissal or suspension from a job, decreasing income, and difficulties in a relationship with co-workers. Spiritual pain could be described as a feeling of meaninglessness of life, loss of identity and worthlessness of living [14]. Examples of questions were: “Do you have any distress derived from loss of the future, loss of others, and loss of autonomy?” Responses such as “therapy and other efforts are meaningless since I am dying.”, “My life is empty.”, “I do not have a vivid sensation of living.”, “I am lonely.”, “No one understands my real feelings.”, or “My presence is a nuisance for my family and friends so I want to die soon.” in medical charts were counted as spiritual pain. Sexuality issues contained sexual dysfunction, infertility and decreasing libido.

### Consent

Our study was approved by the ethical committee of Kinki University of Medicine (No 27-185). Since this study was a retrospective observation study analyzing data extracted during routine clinical practice, written informed consent was not necessary according to the ethics guidelines for epidemiological studies developed by the Japanese Ministry of Labor, Health, and Welfare. We paid attention not to infringe on patient privacy as much as possible and publicly displayed information regarding this study on the homepage of our department (http://www.kindai-psychosomatics.com/) so that those unwilling to participate could contact us to refuse participation.

### Measurements

This was a mixed-method statistical study with cancer patients. Chi-square test and Fisher’s exact test were performed to provide a preliminary comparison of the cancer distress types of male and female patients. Analysis to determine the correlation coefficient between each item of distress was performed using Pearson’s analysis. The index for each item is yes = 1 and no = 0. For gender it is male = 1, female = 0. Multiple logistic regression analysis was performed to identify the factors associated with gender, using each item of a distress type as an independent variable. All statistical analyses were conducted using SPSS software (version 19.0; SPSS Japan Inc., Tokyo).

## Results

### Patient characteristics

Detailed demographic and clinical characteristics of the patients are listed in Table 1. The data of 101 cancer patients were analyzed and the most common cancer site was breast cancer. This table demonstrated that there were more female patients than male patients (female: male ratio = 71:30). Characteristics were assessed at the time of the first visit to our department. Although there were no significant differences, more than half of the female patients came to our psycho-oncology outpatient service within 3 months after diagnosis. Conversely, more than half of the male patients came after 3 months. Patients who had not undergone therapy came to our department just after diagnosis. Approximately two thirds of both female and male patients had a history of operation and chemotherapy, however, female patients experienced hormonal therapy much more than male patients.

**Table 1:** Demographic and clinical characteristics of patients

	Female	Male
*N*	71	30
Age (years)	55.7 ± 12.5	64.1 ± 11.8
Primary cancer site		
Breast	50	0
Digestive organ	4	14
Lung	6	6
Head & Neck	3	5
Gynecological organ	5	0
Urological organ	1	3
Blood	0	2
Others	1	1
Recurrence	18	11
Periods after diagnosis^a^		
< 1 month	8	2
≥ 1–3 months	39	7
≥ 3–6 months	21	13
≥ 6 months	3	8
Profiles of therapies^b^		
None	8	1
Operation	54	23
Chemotherapy	48	24
Radiation	31	8
Hormonal therapy	33	1

Data are shown as a number and Age is described as mean ± SD

^a^Periods after first diagnosis or recurrence

^b^The sum exceeds the patient total number because one patient experienced additional therapy

### The gender differences in the cancer patient distress types

In the chi-square test and Fisher’s exact test shown in Table 2, female patients were more likely to be suffering from psycho-social issues such as changes in appearance, family problems, and sexuality issues than male patients. Male patients were more likely to have spiritual pain.

**Table 2:** The association of gender and distress type

	Male (*N* = 30)	Percent	Female (*N* = 71)	Percent	*p* Value
Pain	17	56.7	31	43.7	0.2317
Changes in appearance	2	0.7	17	23.9	0.0423*
Anxiety	23	76.7	54	76.1	0.9475
Depressive mood	12	40.0	27	38.0	0.8525
Family problems	11	36.7	49	69.0	0.0025**
Job-related problems	10	33.3	16	22.5	0.2567
Spiritual pain	15	50.0	16	22.5	0.0062**
Sexuality issues	1	3.3	13	18.3	0.0387*

Results of chi-square test/Fisher’s exact test

*p < 0.05

**p < 0.01

The results of the correlation matrix table are shown in Table 3. Changes in appearance, family problems, spiritual pain, and sexuality issues were significantly correlated with gender. Using yes = 1, no = 0, male = 1, and female = 0, a Pearson’s correlation coefficient value of −0.202 between gender and changes in appearance showed that female patients were more likely to be suffering from changes in appearance (*p* < 0.05). Similarly, female patients were more likely to be suffering from family problems (*p* < 0.01) and sexuality issues (*p* < 0.05). Male patients were more likely to be suffering from spiritual pain (*p* < 0.05). Changes in appearance and sexuality issues (*p* < 0.05), anxiety and job-related problems (*p* < 0.01), depression and family problems (*p* < 0.05), and family problems and sexuality issues (*P* < 0.05) were correlated with each other in this model. No other correlations were found.

**Table 3:** The correlation between each distress type

		Gender	Pain	Changes in appearance	Anxiety	Depression	Family problems	Job-related problems	Spiritual pain	Sexuality issues
Gender	Pearson’s correlation coefficient									
	Significance probability (both sides)									
	*N*	101								
Pain	Pearson’s correlation coefficient	0.119								
	Significance probability (both sides)	0.236								
	*N*	101	101							
Changes in appearance	Pearson’s correlation coefficient	−0.202	0.049							
	Significance probability (both sides)	0.043 ^a^	0.625							
	*N*	101	101	101						
Anxiety	Pearson’s correlation coefficient	0.007	−0.028	0.150						
	Significance probability (both sides)	0.948	0.784	0.135						
	*N*	101	101	101	101					
Depression	Pearson’s correlation coefficient	0.019	0.019	−0.700	0.108					
	Significance probability (both sides)	0.854	0.851	0.489	0.281					
	*N*	101	101	101	101	101				
Family problems	Pearson’s correlation coefficient	−0.301	−0.021	0.140	−0.083	0.200				
	Significance probability (both sides)	0.002 ^b^	0.837	0.163	0.412	0.045 ^a^				
	*N*	101	101	101	101	101	101			
Job-related problems	Pearson’s correlation coefficient	0.113	0.029	0.180	0.276	0.184	0.026			
	Significance probability (both sides)	0.261	0.772	0.071	0.005 ^b^	0.065	0.800			
	*N*	101	101	101	101	101	101	101		
Spiritual pain	Pearson’s correlation coefficient	0.272	0.097	−0.046	0.119	0.134	−0.018	0.148		
	Significance probability (both sides)	0.006 ^b^	0.332	0.650	0.235	0.183	0.857	0.139		
	*N*	101	101	101	101	101	101	101	101	
Sexuality issues	Pearson’s correlation coefficient	−0.198	0.020	0.247	0.157	0.035	0.215	0.091	−0.143	
	Significance probability (both sides)	0.047 ^a^	0.844	0.013 ^a^	0.118	0.729	0.031 ^a^	0.363	0.155	
	*N*	101	101	101	101	101	101	101	101	101

Analysis to determine the correlation coefficient for each item of distress was performed using Pearson’s analysis. The item indexes are yes = 1, no = 0, male = 1, and female = 0

^a^Correlation coefficient is significant at the 5 % level (both sides)

^b^Correlation coefficient is significant at the 1 % level (both sides)

In the multiple logistic regression model, family problems were significantly associated with gender (odds ratio, 0.27; *CI*_95_ = 0.10–0.71; *p* < 0.01), as was spiritual pain (odds ratio, 3.35; *CI*_95_ = 1.27–8.83; *p* < 0.05). No other associations were found.

## Discussion

### Patient characteristics

More than twice the number of female than male cancer patients had asked for psycho-oncological support and come to our department. One of the reasons for this feature seems to be that breast cancer patients make up about 50 % of the total patients in this study. The reason female patients experienced hormonal therapy much more than male patients was because of the high number of breast cancer patients.

### Gender differences of cancer distress

The chi-square and Fisher’s exact tests showed an association of female patients with changes in appearance, family problems, and sexuality issues and of male patients with spiritual pain. Pearson’s analysis showed the same results and changes in appearance and sexuality issues, anxiety and job-related problems, depression and family problems, and family problems and sexuality issues were correlated. However, in the multiple logistic regression model, only family problems and spiritual pain were significantly associated with gender.

#### Changes in appearance

Changes in appearance cause serious distress for cancer patients, which is manifested as five D’s: Death; Dependence on family or medical staff; Disfigurement; Disruption of life, purpose or desire; and Disability [15]. As for women, it is imaginable that cosmetic problems easily affect their psychological state and quality of life. Previous studies showed that many breast cancer patients suffer from psychological distress due to adverse effects and lifelong physical disfigurement [16]. Head and neck cancer patients also face functional impairment and disfigurement caused by cancer and/or its treatment and have psychosocial difficulties such as depressed mood [17]. Colon and bladder stoma is another distress for cancer patients. Not only irreversible changes, but also temporary changes in appearance such as chemotherapy-induced alopecia, also give a psychological burden to cancer patients [18, 19]. Moreover, these changes in appearance might relate to sexuality issues [20, 21], and there was a significant correlation in Pearson’s analysis in this study.

Cancer patients are often reluctant to discuss these issues with their healthcare team, so more effective screening scales [22, 23], information, education [24] and care are needed [11]. Previous studies showed that social support and self-efficacy mediate the relationship between social distress and emotional distress in head and neck cancer patients with disfigurements [25, 26].

#### Sexuality issues

Sexual dysfunction is one of the most common and distressing consequences of cancer treatment [27] and this often occurs in patients with breast cancer and gynecological cancers [28]. The distress of sexuality issues consists of the complicated combination of physical effects of cancer treatment, women’s intrapsychic experiences of changes to sexuality and her relationship with her partner, and the role of gendered discourse [29]. In this study, the number of gynecological cancer patients was small, but they seemed to suffer the same type of problems as those of breast cancer patients.

Considering the combined factors of sexuality issues, in order to improve the sexual dysfunction of breast cancer patients following mastectomy, breast reconstruction and a reciprocal communication style were important for couples’ coping [30]. The correlation between sexuality issues and family problems was also significant in Pearson’s analysis in this study. Several Japanese women hesitate to talk about sexual problems with their partners. In Japanese culture, there exists a way of thinking that breasts and the uterus are symbols of femininity. When women have a mastectomy or hysterectomy, they seem to think of this situation as the loss of femininity and may lose self-esteem. However, this tendency is not only seen in Japan, but is also common in other countries [31]. Cancer patients need more open-minded discussion and support from their partners and healthcare professionals [32]. The approach from nurses [33] and web-based support groups might be feasible due to their anonymity [34], and health care providers should give more distress screening, information and treatment interventions to cancer patients with sexuality distress [35, 36].

#### Family problems

Female cancer patients had more psycho-social distress regarding family problems than males.

Among family members, the relationship with a partner is crucial. For example, the provision or withdrawal of a partners’ support can have a considerable impact on the psychosocial adjustment of female colorectal cancer patients with ostomies [37]. Furthermore, the relationship with their partner is related to their sexuality. Previous studies showed that the psychosocial distress of breast cancer patients is related to their relationships and the adaptation of their partners [38]. Another study showed that not only female cancer patients, but also female partners of male cancer patients, perceived more psychological distress and a lower quality of life than women in healthy couples, although male partners of female cancer patients did not differ from their healthy controls [9]. For example, spouses of prostate cancer patients suffered conditions affecting their mood, mental and physical health, and sexual function [10].

In addition, many Japanese women play an important role as a caregiver for their children and parents. As Japan is leaning toward an aging society with fewer children, family care is becoming important and the family function might be decreasing. Female patients need more psychological support from their family members, and social support for cancer patients is also vital.

#### Spiritual pain

Male patients were more likely to have spiritual pain than female patients in this study. Previous studies have not mentioned the exact reason for this correlation. In the detailed demographic and clinical characteristics of the patients in Table 1, male patients came to the psycho-oncology outpatient service later than female patients, although there was no statistically significant difference. One of the reasons for this delay might be related to a hesitation by male patients to express their distress. Another is that female patients might react with early stage psychological distress such as anxiety and/or depression after hearing their diagnosis, while male patients might react with a feeling of worthlessness or loneliness included in spiritual pain. In addition, female patients might be influenced by close relationships with family members and male patients might be interested in the meaning of life. Further investigation of the relation between the period when patients come to the outpatient service and the type of distress and individual case scrutiny are needed in the future.

In summary, four items of cancer distress were related to gender differences in this study. Previous studies showed that women suffered from more appearance-related symptoms and distress resulting from chemotherapy than men [11]. The same findings that female patients were more likely to be suffering from changes in appearance were found in our study. In addition, many previous studies discussed the sexual dysfunction of patients with breast cancer and gynecological cancers [28], and our study also revealed that female patients were more likely to be suffering from sexuality issues than were male patients. However, the novelty of this study is that it clarified the association of family problems with female patients and the association of spiritual pain with male patients.

The present study has several limitations. First, this study was based on consultation cases only in our hospital and the female: male patient ratio was not equivalent. Second, the number of breast cancer patients was large, so it might bring bias to our statistical results, for example, the significant association of gender with sexuality issues. Third, this study was performed by extracting all the items from the patients’ medical charts and assessment by several doctors, therefore, a possibility of assessment bias exists.

Although our study has several limitations, some highly suggestive results are seen as helpful for clinical psycho-oncology practice and for suggesting future studies. In order to elucidate the significance of a specific gender-based support for cancer patients, further research addressing the present study’s limitations is necessary.

## Conclusions

There were gender differences in the distress types of cancer patients. Female cancer patients were more likely to be suffering from psycho-social issues such as changes in appearance, family problems, and sexuality issues than were male patients, and male patients were more likely to have spiritual pain. In order to improve the quality of whole cancer care, more intensive intervention by medical professionals and social support is needed from the viewpoint of gender-based medicine and psycho-oncology.

## Abbreviations

HADS: hospital anxiety depression scale
NRS: numeric rating scale
